# Supplementary material for: Advancing Digital Precision Medicine for Chronic Fatigue Syndrome through Longitudinal Large-Scale Multi-Modal Biological Omics Modeling with Machine Learning and Artificial Intelligence
Source: ArXiv. 2025 Jun 18:arXiv:2506.15761v1. Preprint. [Version 1] (PMC12447721)
Supplement: Supplement 1 [file NIHPP2506.15761v1-supplement-1.pdf]

Supplemental Figure

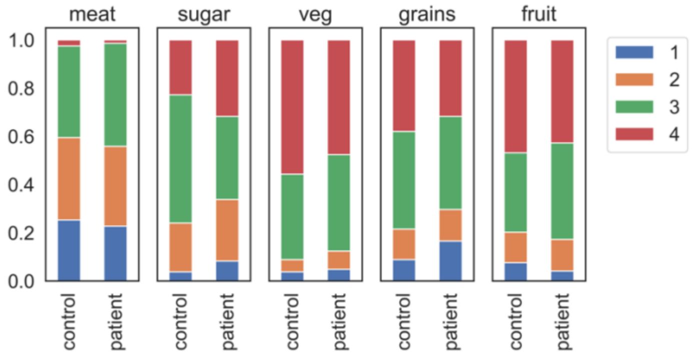

Supplemental Figure 1. The dietary distributions of control and patient cohorts. The dietary questionnaire was summarized into five categories and their frequency in the last week. Categories: Meat - red meat; sugar - desserts, sweets, soda, or juice; veg – fresh vegetables; grains, whole grains (e.g., oatmeal, quinoa, whole wheat products); fruit - fresh fruit. Frequency: 1, Never; 2, Once; 3, 2 to 5 times; 4, Daily.

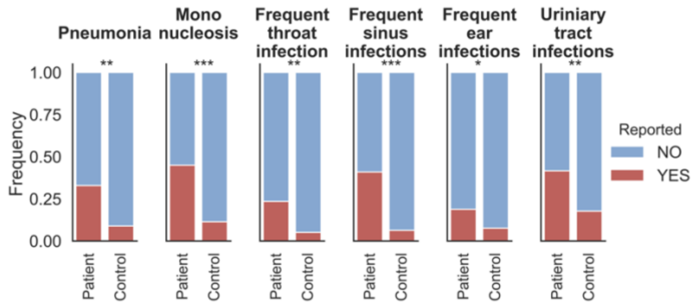

Supplemental Figure 2. ME/CFS patients reported significantly more infection-related histories. More than 30% ME/CFS patients reported pneumonia, mononucleosis, frequent sinus infections and urinary tract history. The p value was computed by Chi-squared test. p-values annotations: *: 0.01 < p <= 0.05, **: 0.001 < p <= 0.01, ***: 1e-04 < p <= 0.001

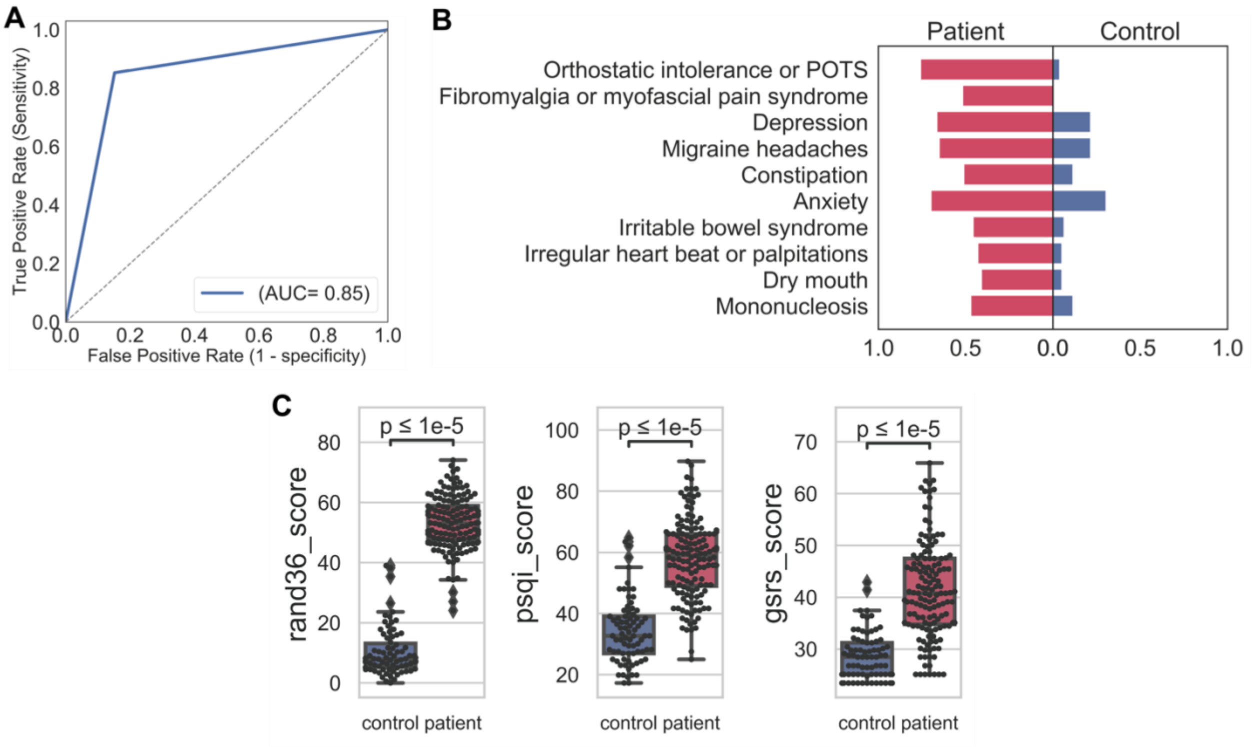

Supplemental Figure 3. The host phenotype in ME/CFS. A) The performance of the naïve Bayesian classification model based on medical history records to identify clinical features that discriminate healthy controls vs. patients. The area under the curve, AUC = 0.85. B) Top ten predicted features and their probabilities to discriminate both cohorts were presented in separate directions on the x-axis. For each feature, the probability of experiencing the symptom in the patients was presented to the left on the x-axis and the probability in the controls was presented to the right. C) Based on our scoring system, patients had significantly more anomalous mental and physical health conditions identified by higher rand36, Pittsburgh sleep quality, and gastrointestinal symptom rating scale scores (Table S2). p-values were computed by Wilcoxon rank-sum test.

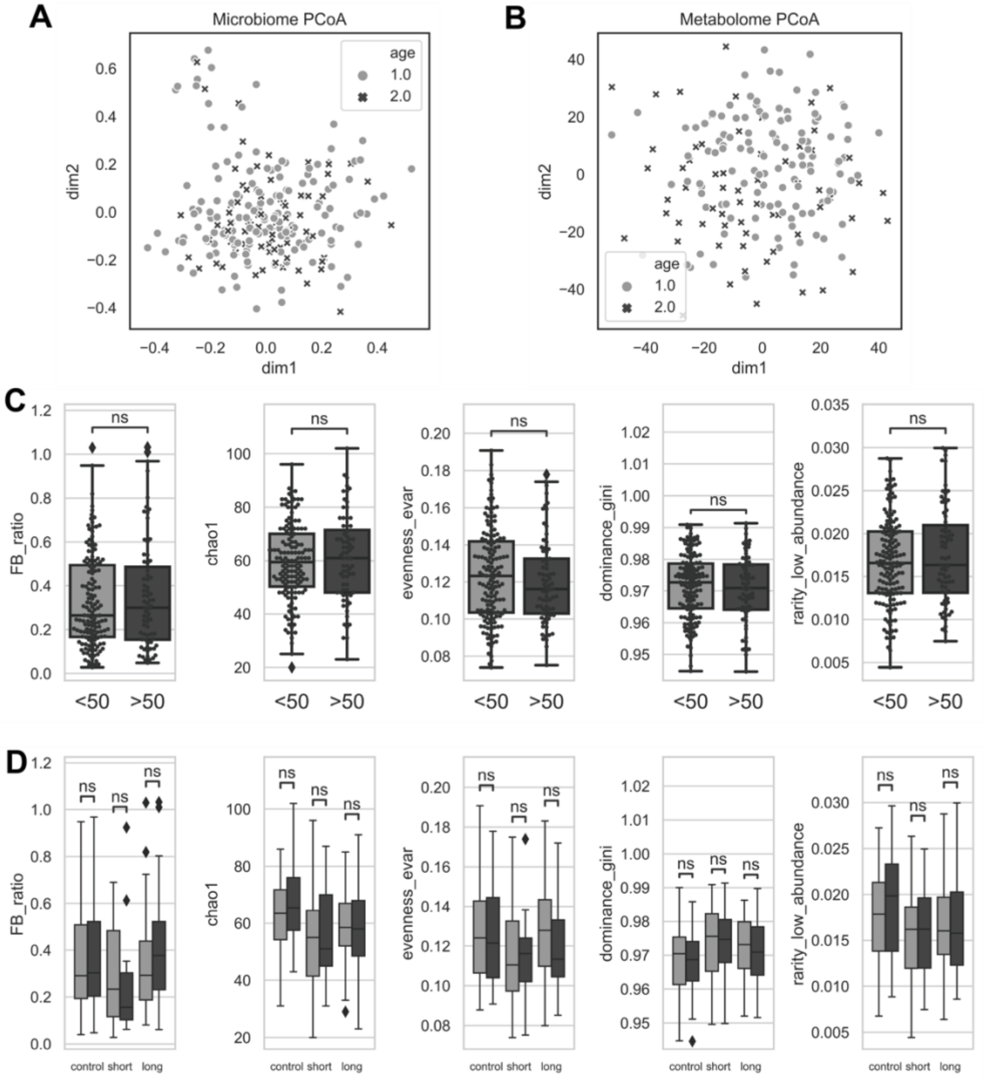

Supplemental Figure 4. Age is not a significant confounder of the gut microbiome and plasma metabolome. A) The Principal Correspondence Analysis (PCoA) based on gut microbiome-derived Bray-Curtis dissimilarity distance. The contribution of age was not significant with a p-value > 0.05 (PERMANOVA, see Methods). B) Principal Correspondence Analysis (PCoA) based on normalized plasma metabolome profile. In C) and D), microbial community structure was not significantly different between young (<50 years old) and old (>=50 years old) in C) all cohorts and D) cohort by disease stage (control, short-term, and long-term), respectively. Community structure was indicated by FB_ratio (Firmicutes:Bacteroides ratio), Chao 1 index (richness), evenness_evar (Smith and Wilson's Evar index), dominance_gini (Gini index of the

dominant species >0.2% relative abundance), and rarity_low_abundance (proportion of the least abundant species <0.2% relative abundance). p-values were computed by Wilcoxon rank-sum test. ns, not significant.

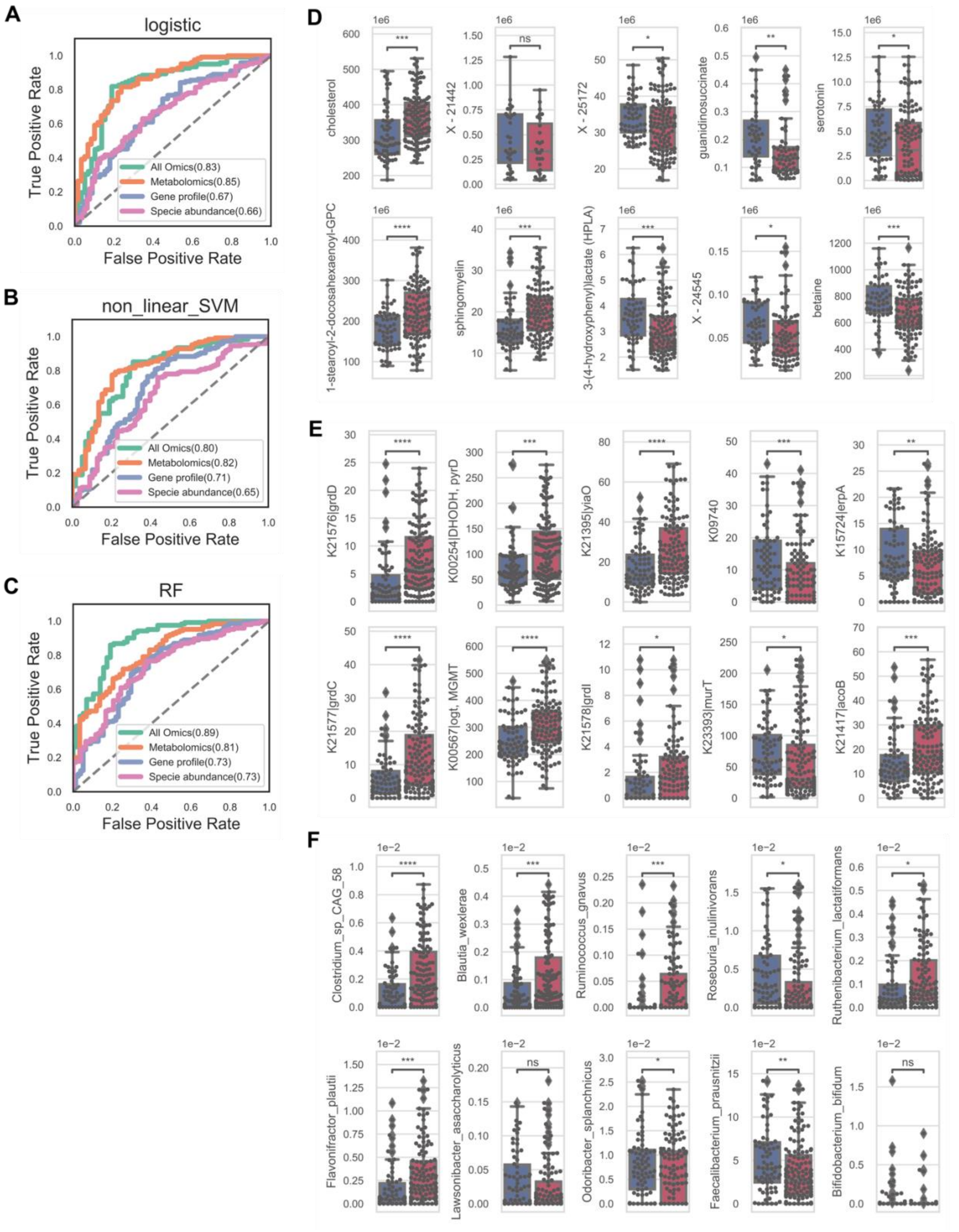
A
logistic
True Positive Rate
False Positive Rate
All Omics(0.83)
Metabolomics(0.85)
Gene profile(0.67)
Specie abundance(0.66)
B
non_linear_SVM
True Positive Rate
False Positive Rate
All Omics(0.80)
Metabolomics(0.82)
Gene profile(0.71)
Specie abundance(0.65)
C
RF
True Positive Rate
False Positive Rate
All Omics(0.89)
Metabolomics(0.81)
Gene profile(0.73)
Specie abundance(0.73)
D
cholesterol
X - 21442
X - 25172
guanidinosuccinate
serotonin
1-stearoyl-2-docosahexaenoyl-GPC
sphingomyelin
3-(4-hydroxyphenyl)lactate (HPLA)
X - 24545
betaine
1e6
E
K21576|grdD
K00254|DHODH, pyrD
K21395|yiaO
K09740
K15724|erpA
K21577|grdC
K00567|ogt, MGMT
K21578|grdI
K23393|murT
K21417|acoB
F
Clostridium_sp_CAG_58
Blautia_wexlerae
Ruminococcus_gnavus
Roseburia_inulinivorans
Ruthenibacterium_lactatiformans
Flavonifractor_plautii
Lawsonibacter_asaccharolyticus
Odoribacter_splanchnicus
Faecalibacterium_prausnitzii
Bifidobacterium_bifidum
1e-2
ns
*
**
***
****

Supplemental Figure 5. Multi-‘omics models to classify the onset of ME/CFS (control vs. patient). A-C) Performance of the classifiers using area under the curve (AUC) was evaluated using 10 randomized and 10-fold cross-validations for each model: LASSO logistic regression, Support vector machine (SVM) and random-forest (RF) models. Models were designed based on species relative abundance (pink), KEGG gene profile (blue) or plasma metabolome (orange) individually, or a taken altogether (‘omics green) with top 30 features from three individual models (see Methods). D-F) Discriminant features identified from gradient boosting classifiers significantly changed in the patient cohort compared to the healthy individuals. p-values were computed by Wilcoxon signed-rank test. p-value annotation legend: ns: p > 0.05, *: 0.01 < p <= 0.05, **: 0.001 < p <= 0.01, ***: 1e-04 < p <= 0.001, ****: p <= 1e-04.

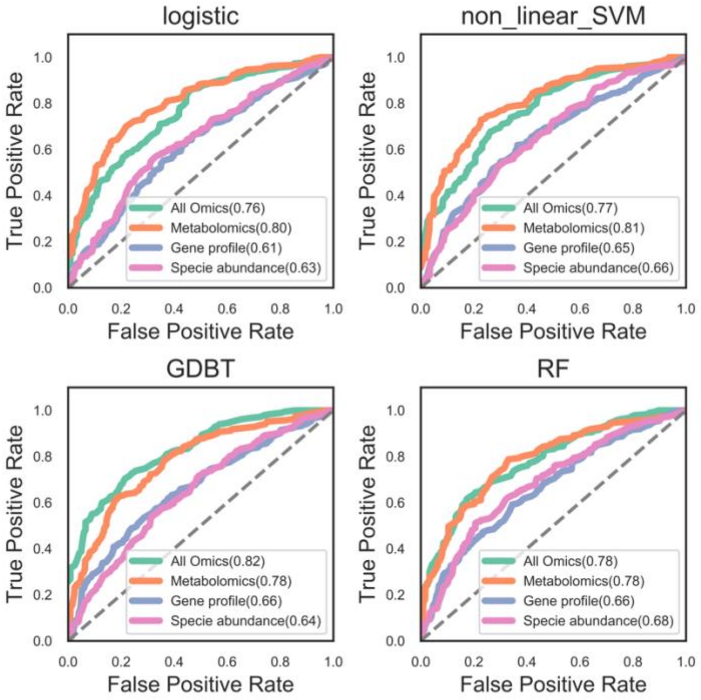

Supplemental Figure 6. Multi-'omics models to classify the duration of ME/CFS (control vs. short-term vs. long-term). As above, the performance of the classifiers using the area under the curve (AUC) was evaluated using 10 randomized and 10-fold cross-validations for each model: LASSO logistic regression, support vector machine (SVM), and random forest (RF) models. Models were designed based on species relative abundance (pink), KEGG gene profile (blue), or plasma metabolome (orange) individually, or taken all together ('omics green) with top 30 features from three individual models shown.

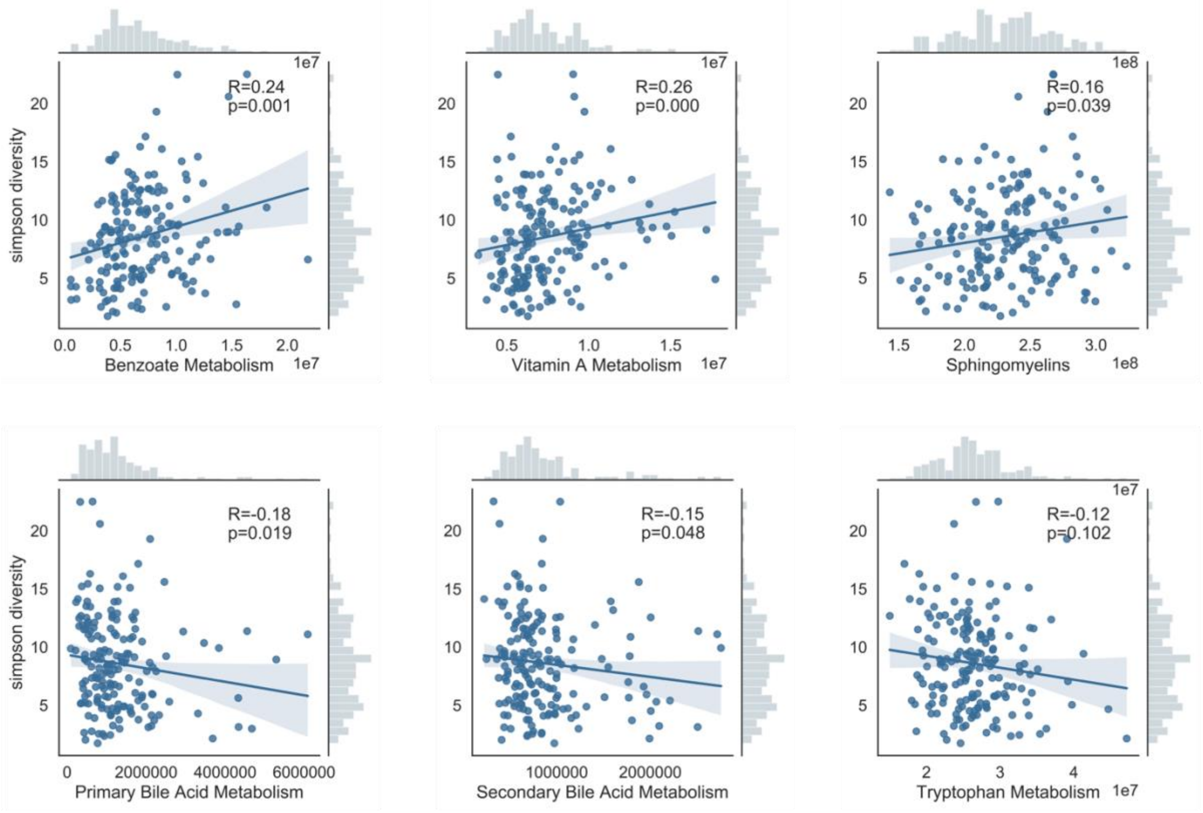

Supplemental Figure 7. Plasma metabolic pathways are correlated with gut community structure in the cohort. The gut microbiome community structure index was indicated by Simpson diversity and the relative abundance of the secondary metabolic pathway was indicated by the mean of all metabolites in the pathway. Each dot indicates one sample, and regression line and the confidence intervals shaded are shown. The data distributions are displayed in the top and right, respectively. The rho value and p-value were computed by the Spearman correlation.

Supplemental Tables

Supplementary Table 1 Demographic information of cohorts and Clinical metadata summary. Related to STAR Methods.

Supplementary Table 2 Metadata. Related to STAR Methods.

Supplementary Table 3 Species relative abundances derived from MetaPhlAn3. Related to Figure 2, 3 and 4.

Supplementary Table 4 KEGG gene reads count from metagenomic data. Related to Figure 4.

Supplementary Table 5 Plasma metabolome raw data (peaks area-under-the-curve). Related to Figure 4 and 5.

Supplementary Table 6 Feature importance and annotation from multi-'omics classification model and Butyrate pathway: the statistics of key microbes and key enzymes in KEGG butanoate metabolism, and their correlations with key plasma metabolites. Related to Figure 5 and 6.

Supplementary Table 7 Confounder driven features identified by MaAsLin2, Related to STAR Methods

Supplemental Figure

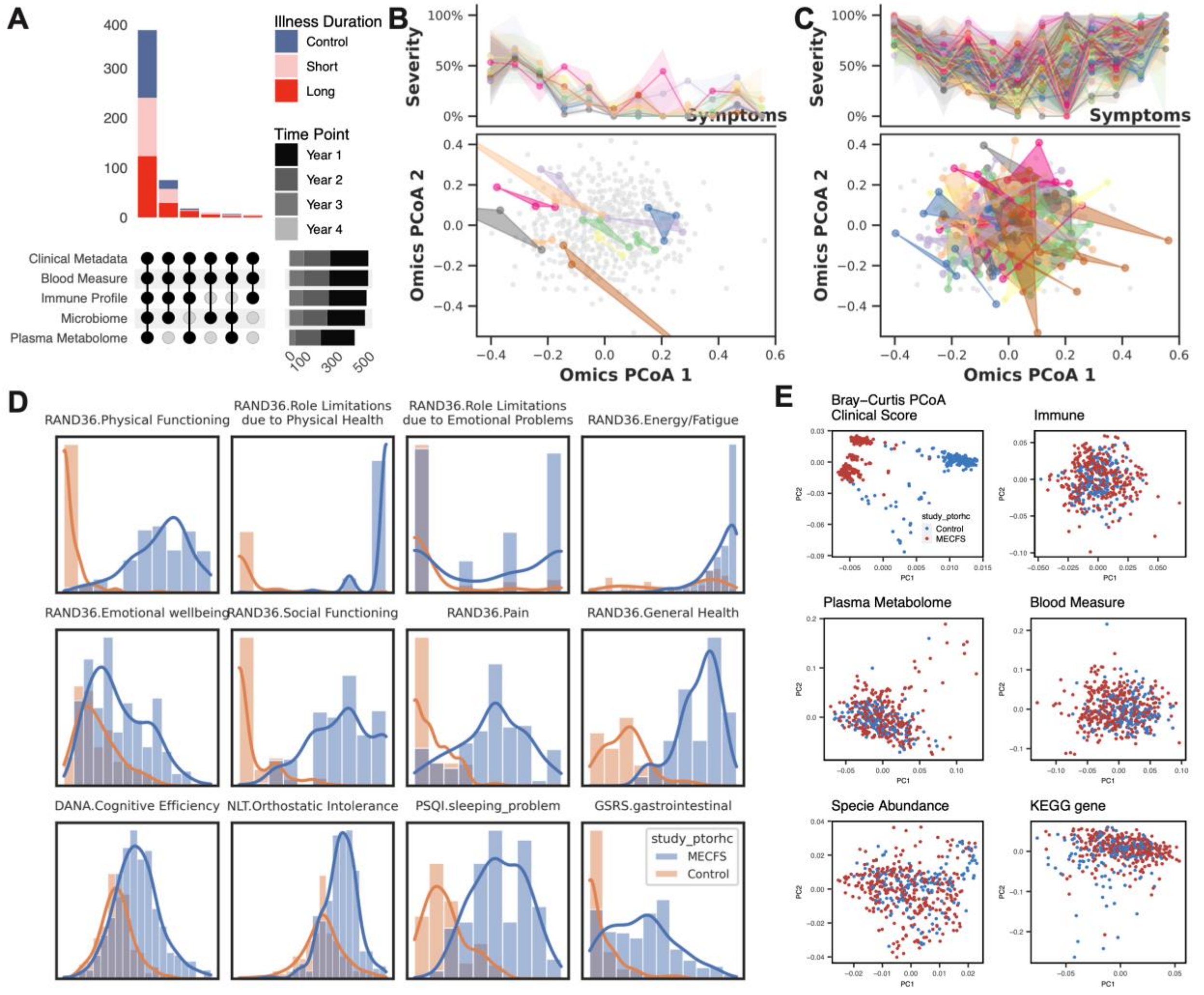

Supplemental Figure 1: Data Pairedness Overview and Heterogeneity in Healthy and Patients.

A) Cohort Composition and Data Collection. Over four years, 515 time points were collected: baseline year from all 249 donors (Healthy N=96, ME/CFS N=153); second year from 168 individuals (Healthy N=58, ME/CFS N=110); third year from 94 individuals (Healthy N=13, ME/CFS N=81); fourth year from N=4 ME/CFS patients. Nearly 400 collection points included complete sets of 5 ‘omics datasets, with others capturing 3-4 ‘omics profiles. Clinical metadata and blood measures were collected at all 515 points. Immune profiles from PBMCs were recorded at 489 points, microbiome data from stool

samples at 479 points, and plasma metabolome data at 414 points. A total of 1,471 biosamples were collected. **B-C) Heterogeneity of B) Healthy Controls and C) All Patients in Symptom Severity and 'Omics Profiles.** Supplemental information for Figure 1B, which shows examples from 20 patients. Variability in symptom severity (top) and 'omics profiles (bottom) for all healthy controls and all patients with 3-4 time points. **D) Distribution of 12 Clinical Symptoms in ME/CFS and Control.** Density plots compare the distributions of 12 clinical scores between control (blue) and ME/CFS patients (orange) with the y-axis representing severity (scaled from 0% to 100%). Clinical scores include RAND36 subscales (e.g., Physical Functioning, Emotional Wellbeing), Cognitive Efficiency from the DANA test, Orthostatic Intolerance from the NLT test, Sleep Problems from the PSQI questionnaire, and Gastrointestinal Symptoms from the GSRS questionnaire. **E) Principal Coordinates Analysis (PCoA) of each 'Omics.** PCoA based on Bray-Curtis distance for clinical scores, immune profiles, plasma metabolome, blood measures, species abundance, and KEGG gene data. Control samples (blue) and ME/CFS patients (red) show distinct clustering. Here, except for the clinical scores, controls are indistinguishable from patients, highlighting the difficulty of building classification models. **Abbreviations:** ME/CFS, Myalgic Encephalomyelitis/Chronic Fatigue Syndrome; PCoA, Principal Coordinates Analysis; RAND36, 36-Item Short Form Health Survey; DANA, DANA Brain Vital; NLT, NASA Lean Test; PSQI, Pittsburgh Sleep Quality Index; GSRS, Gastrointestinal Symptom Rating Scale; KEGG, Kyoto Encyclopedia of Genes and Genomes. **Related to:** Figure 1-2.

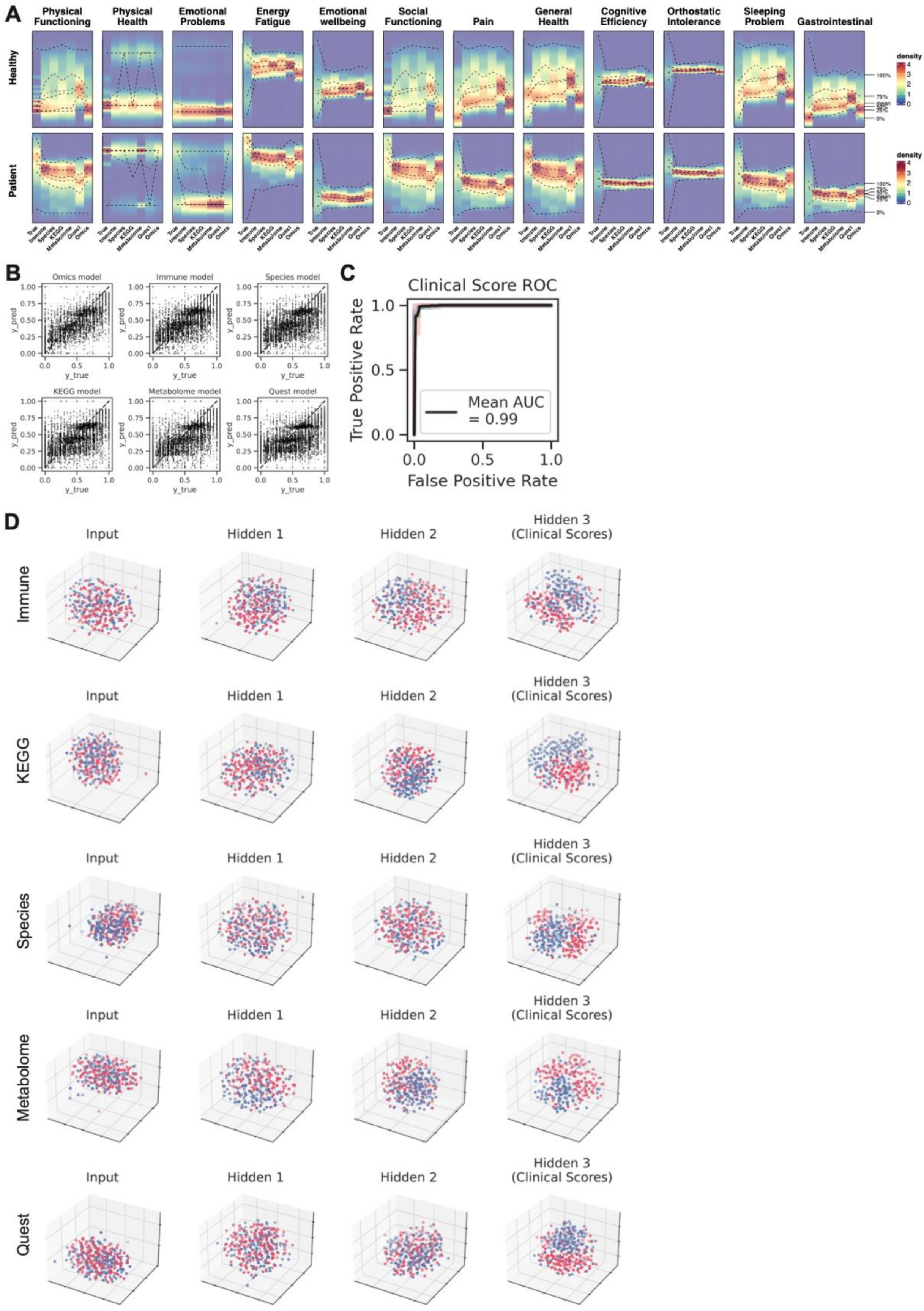
A
Physical Functioning
Physical Health
Emotional Problems
Energy Fatigue
Emotional wellbeing
Social Functioning
Pain
General Health
Cognitive Efficiency
Orthostatic Intolerance
Sleeping Problem
Gastrointestinal
Healthy
Patient
density
100%
75%
mean
50%
25%
0%
True
Immune
Species
KEGG
Metabolome
Quest
Omics
B
Omics model
Immune model
Species model
KEGG model
Metabolome model
Quest model
y_pred
y_true
C
Clinical Score ROC
True Positive Rate
False Positive Rate
Mean AUC = 0.99
D
Input
Hidden 1
Hidden 2
Hidden 3 (Clinical Scores)
Immune
KEGG
Species
Metabolome
Quest

Supplemental Figure 2: BioMapAI's Performance at Clinical Score Reconstruction and Disease Classification. A) Density map of True vs. Predicted Clinical Scores. Supplemental information for Figure 2B, which shows three examples. Here, the full set of 12 clinical scores compares the true score, y (Column 1), against BioMapAI's predictions generated from different 'omics profiles – $\hat{y}_{immune}$, $\hat{y}_{species}$, $\hat{y}_{KEGG}$, $\hat{y}_{metabolome}$, $\hat{y}_{quest}$, $\hat{y}_{omics}$ (Columns 2-7). **B) Scatter Plot of True vs. Predicted Clinical Scores.** Scatter plots display the relationship between true clinical scores (x-axis) and predicted clinical scores (y-axis) for six different models: Omics, Immune, Species, KEGG, Metabolome, and Quest Labs. Each plot demonstrates the clinical score prediction accuracy for each model. **C) ROC Curve for Disease Classification with Original Clinical Scores.** The Receiver Operating Characteristic (ROC) curve evaluates the performance of disease classification using the original 12 clinical scores. The mean Area Under the Curve (AUC) is 0.99, indicating high prediction accuracy, which aligns with the clinical diagnosis of ME/CFS based on key symptoms. **D) 3D t-SNE Visualization of Hidden Layers.** 3D t-SNE plots show how BioMapAI progressively distinguishes disease from control across hidden layers for five trained 'omics models: Immune, KEGG, Species, Metabolome, and Quest Labs. Each plot uses the first three principal components to show the spatial distribution of control samples (blue) and ME/CFS patients (red). The progression from the input layer (mixed groups) to Hidden Layer 3 (fully separated groups) illustrates how BioMapAI progressively learns to separate ME/CFS from healthy controls. **Abbreviations:** ROC, Receiver Operating Characteristic; AUC, Area Under the Curve; t-SNE, t-Distributed Stochastic Neighbor Embedding; PCs, Principal Components; y, True Score; $\hat{y}$, Predicted Score. **Related to:** Figure 2.

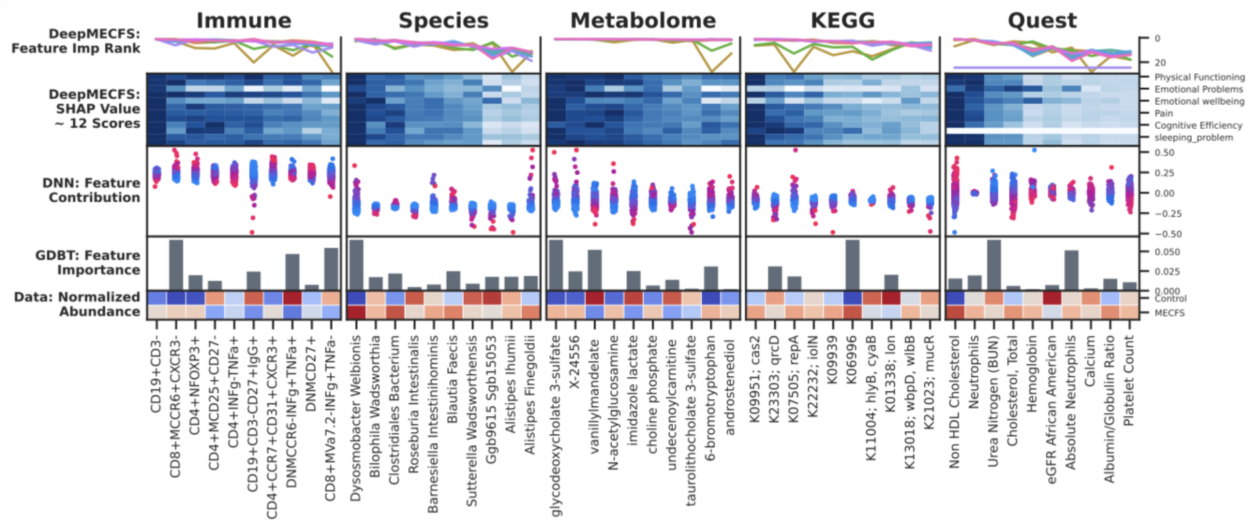

Supplemental Figure 3: Disease-Specific Biomarkers - Top 10 Biomarkers Shared across Clinical Symptoms and Multiple Models. Through the top 30 high-ranking features for each score, we discovered that the most critical features for all 12 symptoms were largely shared and consistently validated across ML and DL models, particularly the foremost 10. Here, this multi-panel figure presents the top 10 most significant features identified by BioMapAI across five 'omics profiles, highlighting their importance in predicting clinical symptoms and diagnostic outcomes across BioMapAI, DNN, and GBDT models, along with their data prevalence. Each vertical section represents one 'omics profile, with columns of biomarkers ordered by average feature importance from right to left. From top to bottom: *1. Feature Importance Ranking in BioMapAI.* Lines depict the rank of feature importance for each clinical score, color-coded by the 12 clinical scores. Consistency among the top 5 features suggests they are shared disease biomarkers crucial for all clinical symptoms; *2. Heatmap of SHAP Values from BioMapAI.* This heatmap shows averaged SHAP values with the 12 scores on the rows and the top 10 features in the columns. Darker colors indicate a stronger impact on the model's output; *3. Swarm Plot of SHAP Values from DNN.* This plot represents the distribution of feature contributions from DNN, which is structurally similar to BioMapAI but omits the third hidden layer (Z^3). SHAP values are plotted vertically, ranging from negative to positive, showing each feature's influence on prediction outcomes. Points represent individual samples, with color gradients denoting actual feature values. For instance, *Dysosmobacteria welbionis*, identified as the most

critical species, shows that greater species relative abundance correlates with a higher likelihood of disease prediction; *4. Bar Graphs of Feature Importance in GBDT*. GBDT is another machine learning model used for comparison. Each bar's height indicates a feature's significance within the GBDT model, providing another perspective on the predictive relevance of each biomarker; *5. Heatmap of Normalized Raw Abundance Data.* This heatmap compares biomarker prevalence between healthy and disease states, with colors representing z-scored abundance values, highlighting biomarker differences between groups. **Abbreviations:** DNN: Here refer to our deep Learning model without the hidden 3, ‘spread out’ layer; GBDT: Gradient Boosting Decision Tree; SHAP: SHapley Additive exPlanations. **Supporting Materials:** Supplemental Table 5. **Related to:** Figure 3.

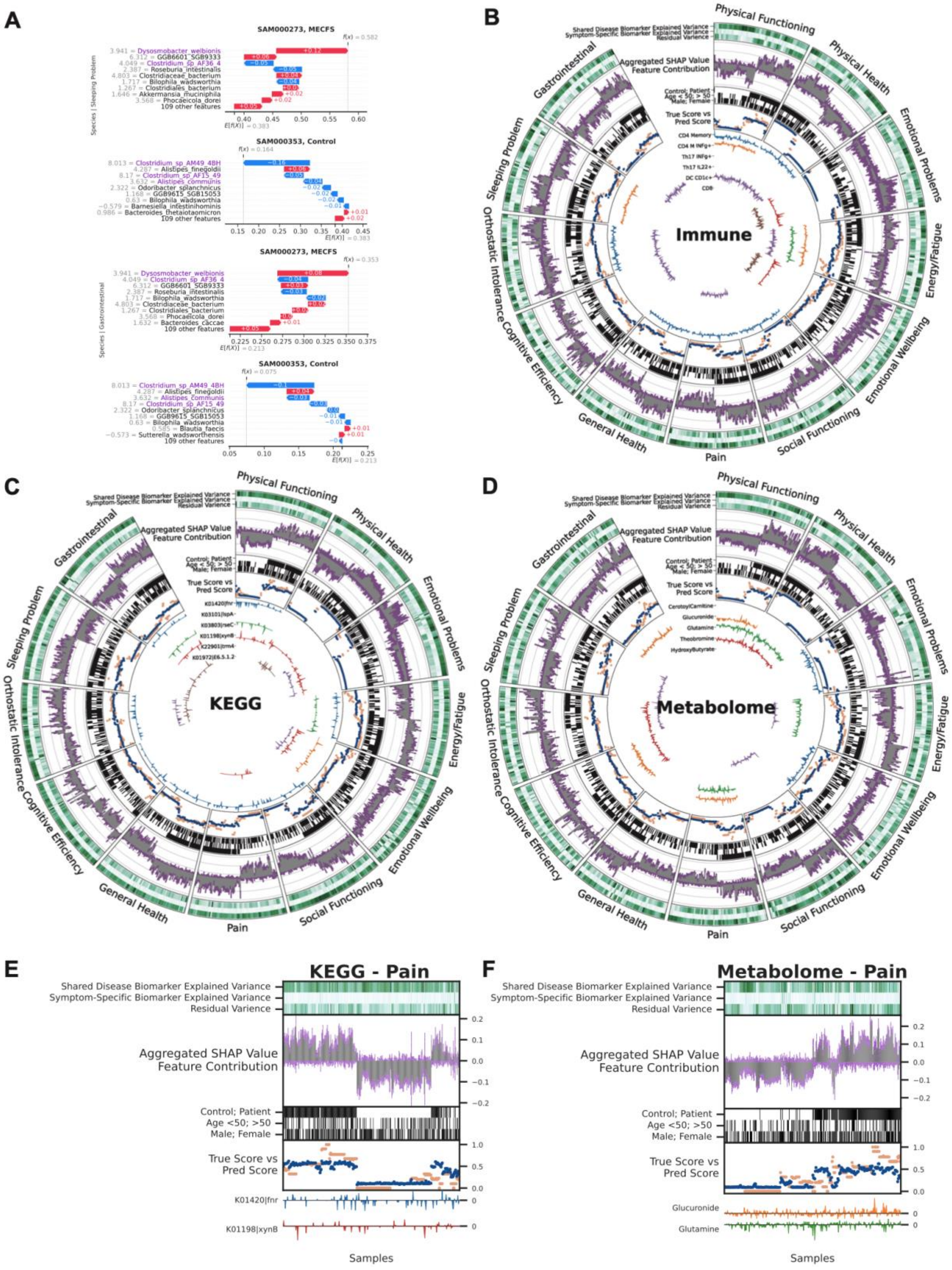

Supplemental Figure 4: Symptom-Specific Biomarkers - Immune, KEGG and

Metabolome Models. By linking ‘omics profiles to clinical symptoms, BioMapAI identified unique symptom-specific biomarkers in addition to disease-specific biomarkers (Supplemental Figure 3). Each ‘omics has a circularized diagram (Figure 3A, Supplemental Figure 4B-D) to display how BioMapAI use this ‘omics profile to predict 12 clinical symptoms and to discuss the contribution of disease- and symptom-specific biomarkers. Detailed correlation between symptom-specific biomarkers and their corresponding symptoms is in Supplemental Figure 5. **A) Examples of Sleeping Problem-Specific Species’ and Gastrointestinal-Specific Species’ Contributions.** Supplemental information for Figure 3D, which shows the contribution of pain-specific species. **B-D) Circularized Diagram for Immune, KEGG and Metabolome Models.** Supplemental information for Figure 3A, which shows the species model. **E-F) Zoomed Segment for Pain in KEGG and Metabolome Model.** Supplemental information for Figure 3B, which shows the zoomed segment for pain in the species and immune models. **Abbreviations and Supporting Materials:** Supplemental Figure 5. **Related to:** Figure 3.

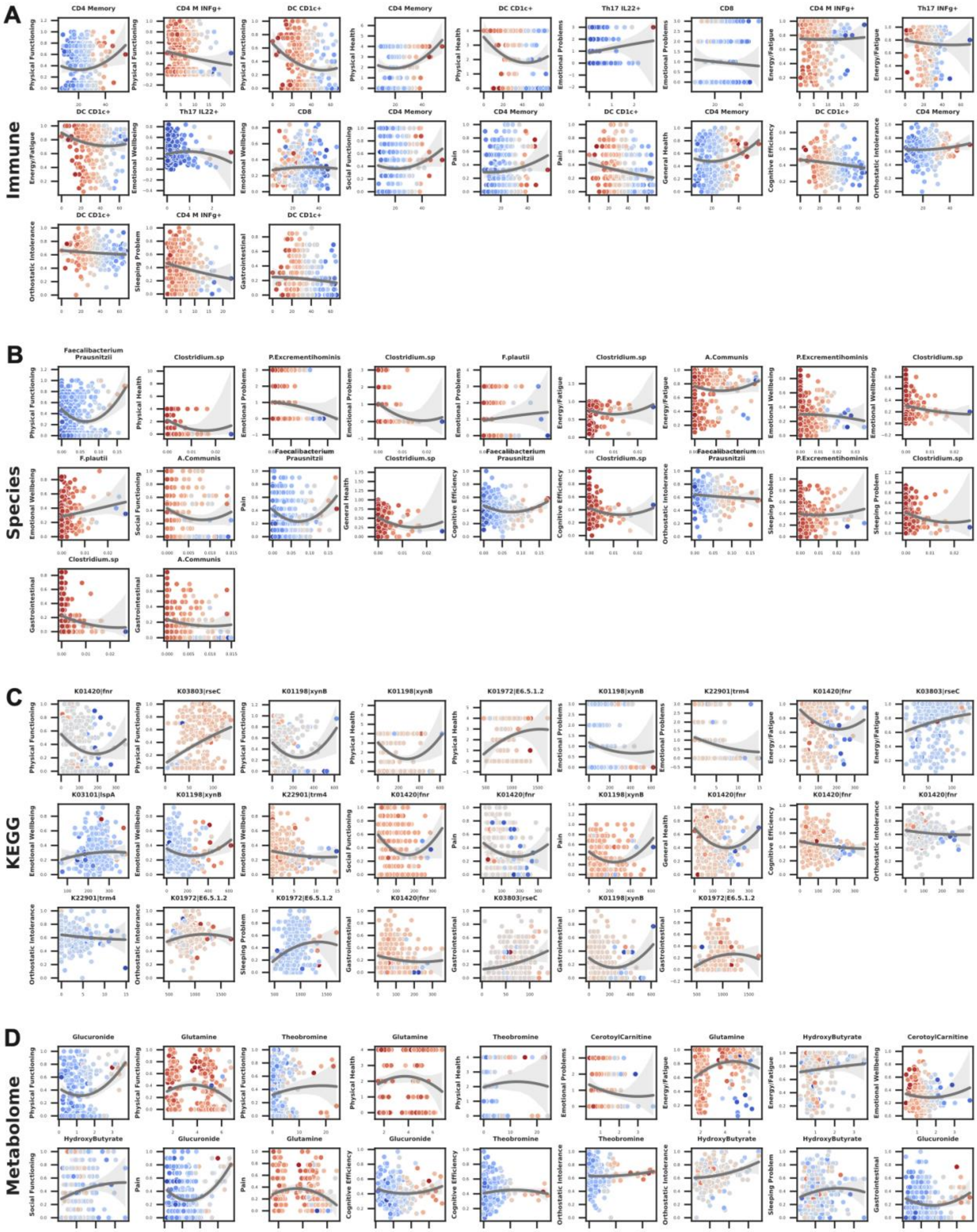

Supplemental Figure 5: Symptom-Specific Biomarkers - Different Correlation Patterns of Biomarkers to Symptom. Supplemental information for Figure 3C, which

shows six pain biomarkers from multiple models. Here for each ‘omics, we plotted the correlation of symptom-specific biomarkers (x-axis) to its related symptom (y-axis), colored by SHAP value (contribution to the symptom). **Abbreviations:** CD4, Cluster of Differentiation 4; CD8, Cluster of Differentiation 8; IFNg, Interferon Gamma; DC, Dendritic Cells; MAIT, Mucosal-Associated Invariant T; Th17, T helper 17 cells; CD4+ TCM, CD4+ Central Memory T cells; DC CD1c+ mBtp+, Dendritic Cells expressing CD1c+ and myelin basic protein; DC CD1c+ mHsp, Dendritic Cells expressing CD1c+ and heat shock protein; CD4+ TEM, CD4+ Effector Memory T cells; CD4+ Th17 rfx4+, CD4+ T helper 17 cells expressing RFX4; *F. prausnitzii, Faecalibacterium prausnitzii; A. communis, Akkermansia communis*; NAD, Nicotinamide Adenine Dinucleotide. **Related to:** Figure 3.

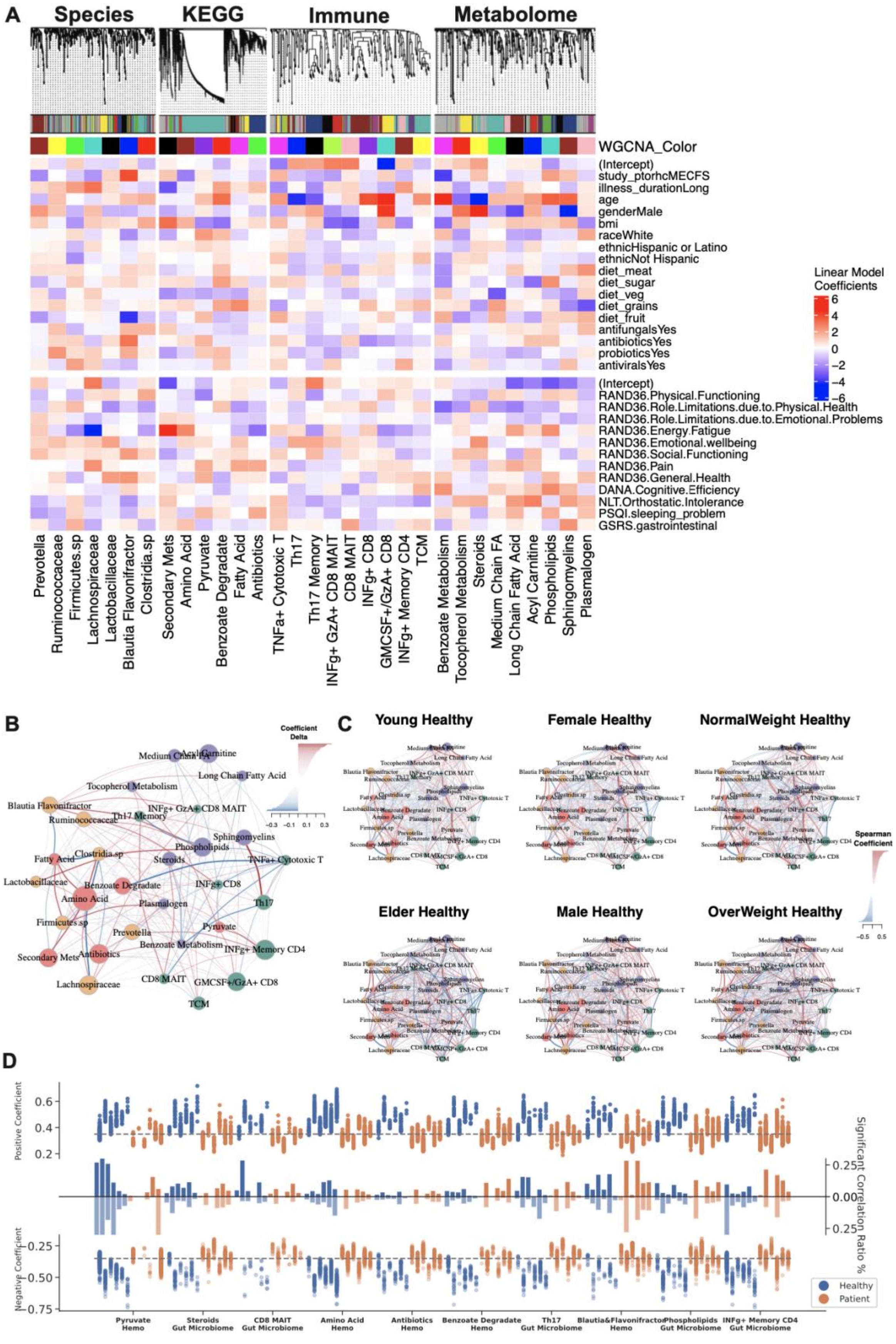
A
Species
KEGG
Immune
Metabolome
WGCNA_Color
(Intercept)
study_ptorhcMECFS
illness_durationLong
age
genderMale
bmi
raceWhite
ethnicHispanic or Latino
ethnicNot Hispanic
diet_meat
diet_sugar
diet_veg
diet_grains
diet_fruit
antifungalsYes
antibioticsYes
probioticsYes
antiviralsYes
(Intercept)
RAND36.Physical.Functioning
RAND36.Role.Limitations.due.to.Physical.Health
RAND36.Role.Limitations.due.to.Emotional.Problems
RAND36.Energy.Fatigue
RAND36.Emotional.wellbeing
RAND36.Social.Functioning
RAND36.Pain
RAND36.General.Health
DANA.Cognitive.Efficiency
NLT.Orthostatic.Intolerance
PSQI.sleeping_problem
GSRS.gastrointestinal
Linear Model Coefficients
6
4
2
0
−2
−4
−6
Prevotella
Ruminococcaceae
Firmicutes.sp
Lachnospiraceae
Lactobacillaceae
Blautia Flavonifractor
Clostridia.sp
Secondary Mets
Amino Acid
Pyruvate
Benzoate Degradate
Fatty Acid
Antibiotics
TNFa+ Cytotoxic T
Th17
Th17 Memory
INFg+ GzA+ CD8 MAIT
CD8 MAIT
INFg+ CD8
GMCSF+/GzA+ CD8
INFg+ Memory CD4
TCM
Benzoate Metabolism
Tocopherol Metabolism
Steroids
Medium Chain FA
Long Chain Fatty Acid
Acyl Carnitine
Phospholipids
Sphingomyelins
Plasmalogen
B
Coefficient Delta
-0.3 -0.1 0.1 0.3
Medium Chain FA
Acyl Carnitine
Long Chain Fatty Acid
Tocopherol Metabolism
Blautia Flavonifractor
Ruminococcaceae
Th17 Memory
INFg+ GzA+ CD8 MAIT
Sphingomyelins
Phospholipids
Fatty Acid
Clostridia.sp
Steroids
TNFa+ Cytotoxic T
Lactobacillaceae
Benzoate Degradate
INFg+ CD8
Amino Acid
Plasmalogen
Th17
Firmicutes.sp
Prevotella
Pyruvate
Benzoate Metabolism
INFg+ Memory CD4
Secondary Mets
Antibiotics
Lachnospiraceae
CD8 MAIT
GMCSF+/GzA+ CD8
TCM
C
Young Healthy
Female Healthy
NormalWeight Healthy
Elder Healthy
Male Healthy
OverWeight Healthy
Spearman Coefficient
-0.5 0.5
D
Positive Coefficient
0.6
0.4
0.2
Significant Correlation Ratio %
0.25
0.00
0.25
Negative Coefficient
−0.25
−0.50
−0.75
Healthy
Patient
Pyruvate Hemo
Steroids Gut Microbiome
CD8 MAIT Gut Microbiome
Amino Acid Hemo
Antibiotics Hemo
Benzoate Degradate Hemo
Th17 Gut Microbiome
Blautia&Flavonifractor Hemo
Phospholipids Gut Microbiome
INFg+ Memory CD4 Gut Microbiome

Supplemental Figure 6: 'Omics WGCNA Modules and Host-Microbiome Network. A) Correlation of WGCNA Modules with Clinical Metadata. Weighted Gene Co-expression Network Analysis (WGCNA) was used to identify co-expression modules for each 'omics layer: species, KEGG, immune, and metabolome. The top dendrograms show hierarchical clustering of 'omics features, with modules identified. The bottom heatmap shows the relationship of module eigengenes (colored as per dendrogram) with clinical metadata – including demographic information and environmental factors - and 12 clinical scores. General linear models were used to determine the primary clinical drivers for each module, with the color gradient representing the coefficients (red = positive, blue = negative). Microbial modules were influenced by disease presence and energy-fatigue levels, while metabolome and immune modules correlated with age and gender. **B-C) Microbiome-Immune-Metabolome Network in B) Patient and C) Healthy Subgroups.** Supplemental information for Figure 4A (Healthy Network) and 4B (Patient Subgroups). Figure 4A is the healthy network; here, Supplemental Figure 6B presented the shifted correlations in all patients. Figure 4B represented the network in patient subgroups; here, Supplemental Figure 6C is the corresponding healthy counterpart, for example, female patients were compared with female controls to exclude gender influences. **D) Differences in Host-Microbiome Correlations between Healthy and Patient Subgroups.** Selected host-microbiome module pairs are grouped on the x-axis (e.g., pyruvate to blood modules, steroids to gut microbiome). Significant positive and negative correlations (top and bottom y-axis) of module members pairs are shown as dots for each subgroup (blue = healthy, orange = patient) (Spearman, adjusted $p < 0.05$), from left to right: Young, Elder, Female, Male, NormalWeight, OverWeight Healthy and Young, Elder, Female, Male, NormalWeight, OverWeight Patient. The middle bars represent the total count of associations. This panel highlights the shifts in host-microbiome networks from health to disease, for example, in patients, the loss of pyruvate to host blood modules correlation and the increase of INFg+ CD4 memory correlation with gut microbiome. **Abbreviations:** WGCNA, Weighted Gene Co-expression Network Analysis; AA, Amino Acids; SCFA, Short-Chain Fatty Acids; IL, Interleukin; GM-CSF, Granulocyte-Macrophage Colony-Stimulating Factor. **Related to**: Figure 4.

Supplemental Table

Supplemental Table 1 Sample Metadata and Clinical Scores

Supplemental Table 2 Model Performance at Reconstructing Twelve Clinical Scores: Averaged Average Mean Squared Error by Model

Supplemental Table 3 Model Performance in Diagnostic Comparison—Within-Cohort, Cross-Validated by Various ML and DL Models

Supplemental Table 4 Model Performance in Diagnostic Comparison—Across Independent Cohorts

Supplemental Table 5 Disease-Specific Biomarker: Averaged Feature Contribution of BioMapAI, DNN and GDBT

Supplemental Table 6 Symptom-Specific Biomarker: Distinct Sets of Biomarkers for Each Symptom

Supplemental Table 7 WGCNA Module Eigengene

Supplemental Table 8 Targeted Pathways: Normalized Gene Read Counts and Their Correlation with Blood Responders
